# From ARB to ARNI in Cardiovascular Control

**DOI:** 10.1007/s11906-016-0694-x

**Published:** 2016-11-11

**Authors:** Estrellita Uijl, Lodi C. W. Roksnoer, Ewout J. Hoorn, A. H. Jan Danser

**Affiliations:** 1 Division of Pharmacology and Vascular Medicine Department of Internal Medicine, Erasmus MC, Rotterdam, The Netherlands; 2 Division of Nephrology and Transplantation, Department of Internal Medicine, Erasmus MC, Rotterdam, The Netherlands

**Keywords:** Diabetes, Neprilysin, Angiotensin, Natriuretic peptide, Chronic kidney disease

## Abstract

Coexistence of hypertension, diabetes mellitus and chronic kidney disease synergistically aggravates the risk of cardiovascular and renal morbidity and mortality. These high-risk, multi-morbid patient populations benefit less from currently available anti-hypertensive treatment. Simultaneous angiotensin II type 1 receptor blockade and neprilysin inhibition (‘ARNI’) with valsartan/sacubitril (LCZ696) might potentiate the beneficial effects of renin-angiotensin-aldosterone inhibition by reinforcing its endogenous counterbalance, the natriuretic peptide system. This review discusses effects obtained with this approach in animals and humans. In animal models of hypertension, either alone or in combination with myocardial infarction or diabetes, ARNI consistently reduced heart weight and cardiac fibrosis in a blood pressure-independent manner. Additionally, LCZ696 treatment reduced proteinuria, focal segmental glomerulosclerosis and retinopathy, thus simultaneously demonstrating favourable effects on microvascular complications. These results were confirmed in patient populations. Besides blood pressure reductions in hypertensive patients and greatly improved (cardiovascular) mortality in heart failure patients, ventricular wall stress and albuminuria were reduced particularly in diabetic patients. The exact underlying mechanism remains unknown, but may involve improved renal haemodynamics and reduced glomerulosclerosis, e.g. related to a rise in natriuretic peptide levels. However, the assays of these peptides are hampered by methodological artefacts. Moreover, since sacubitrilat is largely renally cleared, drug accumulation may occur in patients with impaired renal function and thus hypotension is a potential side effect in patients with chronic kidney disease. Further caution is warranted since neprilysin also degrades endothelin-1 and amyloid beta in animal models. Accumulation of the latter may increase the risk of Alzheimer’s disease.

## Introduction

Cardiovascular diseases cause nearly one third of all deaths worldwide [1], and 50% of these deaths are thought to be directly attributable to hypertension [2]. High blood pressure is an important risk factor in the development of myocardial infarction, hypertensive heart disease, heart failure and stroke. Additionally, hypertension may cause chronic kidney disease (CKD) and end-stage renal disease (ESRD). Patients with CKD are more likely to die from cardiovascular disease than to develop renal failure [3]. They are regarded as a high-risk population within the general hypertensive population. Another high-risk population comprises hypertensive patients with diabetes mellitus. Prevalence of hypertension among diabetic patients is reported in most studies to be greater than 60% and in many studies to be even higher than 75% [4]. Coexistence of hypertension and diabetes synergistically aggravates the risk of developing macro- and microvascular complications [5]. These dismal outcomes emphasize the need for targeted therapy of these vulnerable multi-morbid patient populations.

Treatment of high blood pressure greatly reduces the risk for all-cause mortality, coronary heart disease, heart failure and stroke. Development of renal failure, however, is not prevented by blood pressure lowering, and high-risk populations benefit less from this approach. Indeed, proportional risk reductions for major cardiovascular events are lower or absent in patients with concurrent diabetes or CKD [6]. However, treatment effects vary between different classes of anti-hypertensive drugs. Pharmacological inhibition of the renin-angiotensin-aldosterone system (RAAS) reduces albuminuria and slows the progression of diabetic nephropathy [7]. Unfortunately, effects on cardiovascular events and all-cause mortality remain limited. Initially, it was believed that this was due to incomplete RAAS blockade and RAAS escape phenomena. Although the combination of angiotensin-converting enzyme inhibition (ACEi) and angiotensin receptor blockade (ARB) appears to prevent the development of ESRD more effectively according to one meta-analysis [7], dual therapy is not recommended [8]. The reason is that near complete RAAS suppression does not reduce cardiovascular or all-cause mortality. Yet, it does increase the risk of adverse events, including hypotension, hyperkalaemia and acute kidney injury (AKI) [9]. This most likely relates to the fact that angiotensin II (Ang II) is essential to preserve renal function and glomerular filtration. The kidneys will do everything possible to keep the latter in the normal range, including massive upregulation of renin during RAAS blockade [10]. This mechanism is referred to as the ‘nephrocentric’ reaction to RAAS blockade in patients with heart failure [11]. In exceptional cases, particularly with drugs that accumulate in the kidney (such as renin inhibitors [12]), the nephrocentric reaction may even lead to extrarenal RAAS activation. The 8th Joint National Committee currently recommends ACEi or ARB monotherapy as first-line treatment for patients with CKD [8]. Clearly, rather than blocking the RAAS with two or more drugs, there is a need for interference with alternative systems. This review aims to critically evaluate the potential for cardiovascular control of a new anti-hypertensive treatment strategy, the combination of ARB and neprilysin inhibition: ARNI.

## Mechanism of Action

**Fig. 1:**
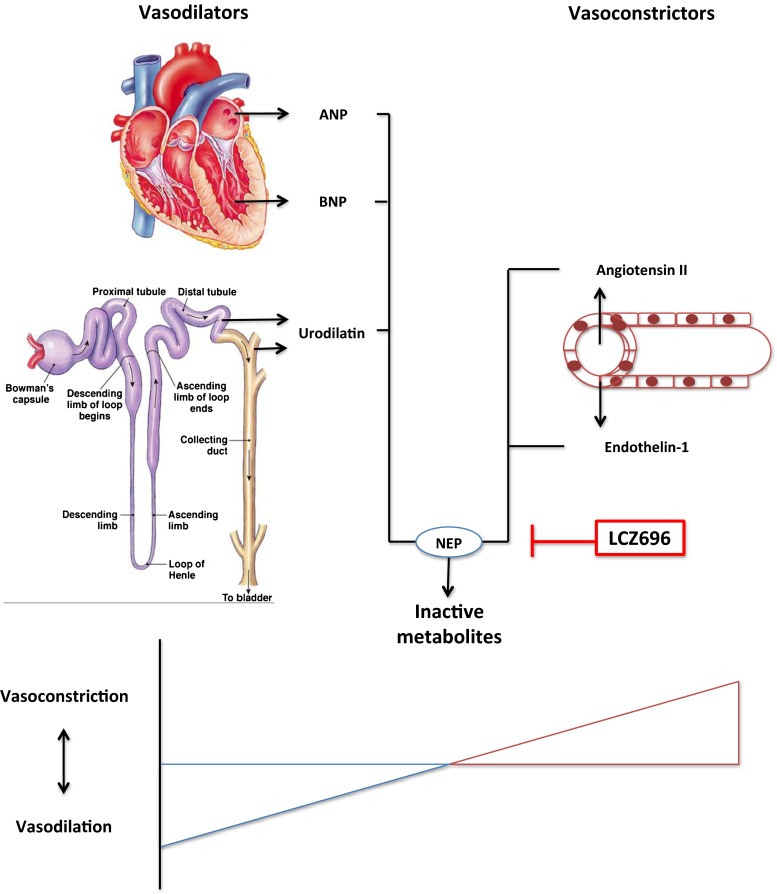
Opposing effects of neprilysin (NEP) inhibition. NEP degrades vasodilators (e.g. A-type (atrial) natriuretic peptide (ANP) produced by atrial myocytes, B-type (brain) natriuretic peptide (BNP) produced by ventricular myocytes and urodilatin produced by the distal convoluted tubule and the collecting duct) as well as vasoconstrictors (e.g. angiotensin II (Ang II) and endothelin-1 produced by endothelial cells) into inactive metabolites. The effect of NEP inhibition with LCZ696 may be unpredictable as it depends on the relative dominance of either vasodilators or vasoconstrictors

Neprilysin, or neutral endopeptidase (NEP), is a transmembrane zinc-dependent metalloprotease with a molecular weight of approximately 85 kDa. Although expressed in many epithelial tissues, its levels are particularly high at the luminal side of the renal proximal tubule [13, 14]. NEP cleaves peptides and thereby inactivates peptide hormones such as glucagon, bradykinin, angiotensin, endothelin-1, substance P, oxytocin, neurotensin, adrenomedullin and natriuretic peptides. Among these are both vasodilators (e.g. bradykinin, natriuretic peptides) and vasoconstrictors (e.g. angiotensin, endothelin-1). Therefore, the effect of NEP inhibition is ambiguous and depends on the relative dominance of the various substrates of NEP (Fig. 1). Consequently, NEP inhibition can even raise blood pressure, as shown in healthy men [15]. It was postulated that the combination of a NEP inhibitor with an ARB would hold the beneficial effects of increasing natriuretic peptides and simultaneously block the harmful effects of (the NEP inhibitor-induced increase in) Ang II. ARBs prevent the binding of Ang II to the Ang II type 1 (AT_1_) receptor, thereby inhibiting Ang II-induced vasoconstriction, aldosterone release, stimulation of the sympathetic nervous system and vascular as well as cardiac remodeling [16]. Natriuretic peptides counter the RAAS not only by inducing natriuresis, diuresis and vasodilation but also by inhibiting renin secretion, via their second messenger cyclic guanosine 3′5′monophosphate (cGMP) [17]. Therefore, reinforcement of the natriuretic peptide system on top of RAAS blockade might have a synergistic effect on blood pressure and cardiac remodeling. LCZ696 (sacubitril/valsartan, brand name Entresto) is the first-in-class ARNI. It is a single molecule in which the ARB valsartan and the NEP inhibitor prodrug sacubitril (AHU377) are combined, in a molar ratio of 1:1 [18]. Sacubitril is almost completely metabolized into its active form LBQ657 (also known as sacubitrilat) via ester hydrolysis, rapidly after ingestion [19]. In humans, sacubitril has a half life of 1.4 h, LBQ657 has a half life of 11.5 h and valsartan has a half life of 9.9 h. LBQ657 is excreted through urine, and the exposure to LBQ657 increases with decreasing renal function [20]. A decrease in renal function does not affect the pharmacokinetics of valsartan, since it is primarily excreted via the biliary route [21].

## Animal Studies

Currently, the number of animal studies using ARNI is limited (Table 1). In 2008, Pu et al. compared the ARB valsartan with the ARNI combination valsartan + CGS25354 and a dual ACE inhibitor/NEP inhibitor in stroke-prone spontaneously hypertensive rats [22]. Compared with valsartan, both ARNI and the dual ACE/NEP inhibitor were more effective in lowering blood pressure and decreasing vascular remodeling and macrophage infiltration. The ACE/NEP inhibitor reduced cardiac hypertrophy more than ARNI, possibly because the ACE/NEP inhibitor also had a greater effect on blood pressure compared with ARNI [22]. Because the study did not include an ACE inhibition-only group, it is unclear if this was due to a difference in effectiveness between ARB and ACE inhibition or if dual ACE/NEP inhibition had a stronger synergistic effect compared with ARNI.

**Table 1:** Comparison of LCZ696 to other anti-hypertensive therapies or vehicle in animal models

Year	Author	Animal model	ARNI (ARB + NEPi)	Comparison	
				ARB	Other
2008	Pu et al.	SHRSP rats	Valsartan + CGS25354	Valsartan	CGS30440^b^
2010	Gu et al.	Beagle dogs and SD rats	Valsartan + sacubitril^a^	–	–
2015	Von Lueder et al.	SD rats	Valsartan + sacubitril^a^	–	Vehicle
2015	Bai et al.	C57BL/6J mice	Valsartan + sacubitril^a^	Valsartan	Vehicle
2015	Roksnoer et al.	TGR (mRen2) rats	Irbesartan + thiorphan	Irbesartan	Thiorphan, vehicle
2016	Suematsu et al.	C57BL/6J mice + STZ diabetes	Valsartan + sacubitril^a^	Valsartan	Vehicle
2016	Roksnoer et al.	TGR (mRen2) rat + STZ diabetes	Irbesartan + thiorphan	Irbesartan	Vehicle

*ACEi* angiotensin-converting enzyme inhibitor, *NEPi* neprilysin inhibitor, *SHRSP* stroke-prone spontaneously hypertensive rats, *SD* Sprague Dawley, *STZ* streptozotocin

^a^LCZ696

^b^Dual ACEi/NEPi

In 2010, the first animal study using the ARNI LCZ696 was published. Gu et al. reported on the pharmacokinetics of LCZ696 in beagle dogs and its pharmacodynamics in rats [18]. Single oral administration of LCZ696 to rats transgenic for human renin and angiotensinogen caused a dose-dependent decrease in mean arterial pressure (MAP) compared to baseline, while it increased plasma ANP concentration and immunoreactivity in Sprague-Dawley (SD) rats chronically infused with exogenous A-type (atrial) natriuretic peptide (ANP) [18]. LCZ696 was not compared with valsartan alone.

Von Lueder et al. compared LCZ696 with vehicle treatment in SD rats after experimental myocardial infarction [23]. As expected, after 4 weeks of treatment, LCZ696-treated rats had a lower blood pressure, a higher cardiac output, a lower heart weight and less cardiac fibrosis compared with vehicle treatment. In vitro, Von Lueder et al. assessed the effects of valsartan, AHU377, LBQ657 or valsartan + LBQ657 on rat neonatal cardiac myocytes and fibroblasts, after Ang II pretreatment. Valsartan and LBQ657 both inhibited Ang II-induced cardiac myocyte hypertrophy when applied separately. When applied simultaneously, valsartan + LBQ657 only outperformed valsartan when a very low dose or a very high dose of valsartan was combined with LBQ657 (fixed dose). Valsartan decreased collagen accumulation in cardiac fibroblasts, while LBQ657 did not affect collagen accumulation. Dual treatment with valsartan + LBQ657 consistently inhibited Ang II-induced collagen accumulation more than valsartan alone [23]. Unfortunately, in this study, LCZ696 treatment was not compared with valsartan in vivo.

Bai et al. pretreated mice with LCZ696, valsartan or vehicle before inducing ischaemic brain damage by middle cerebral artery occlusion [24]. Despite the absence of a blood pressure difference between the three groups, LCZ696-treated mice had a smaller ischaemic area compared with valsartan-treated mice. LCZ696 caused a significant rise in serum ANP levels, compared with valsartan. Both valsartan and LCZ696 induced an increase in serum renin activity and serum Ang II concentration [24]. However, when calculating the dose of LCZ696, the authors used equal weight ratios instead of equal molar ratios and did not take in account area under the curve data to provide similar exposure to valsartan [18, 25]. The authors did not comment on the absence of a difference in blood pressure between the three treatment groups.

Suematsu et al. used tenfold higher dosages of valsartan and LCZ696, compared with Bai et al., in streptozotocin-treated diabetic mice, after myocardial reperfusion injury [26]. At the end of the 4-week treatment period, only valsartan-treated animals had a significantly lower blood pressure than vehicle-treated animals. Both valsartan- and LCZ696-treated animals had a lower heart weight/body ratio than vehicle-treated animals. LCZ696-treated animals displayed a better ejection fraction, less cardiac fibrosis and lower cardiac TGF-β and ANP expression compared with vehicle-treated animals. How exactly LCZ696 exerted its beneficial effects on TGF-β, fibrosis and cardiac function, independently from blood pressure, remained unclear [26].

In a recent study by our group, ARB (irbesartan) and ARNI (irbesartan + NEP inhibitor thiorphan) were compared in diabetic, hypertensive rats. ARB and ARNI induced a similar blood pressure-lowering effect, but ARNI-treated animals displayed a lower heart weight, less proteinuria and less focal segmental glomerulosclerosis as compared to ARB-treated animals [27]. When analysing the eyes of these diabetic animals, ARNI-treated animals also had less severe diabetic retinopathy [Qiuhong Li, University of Florida, Gainesville, USA, unpublished observations].

This led to the conclusion that ARNI exerts beneficial effects in the heart, kidney and eye independent of its blood pressure-lowering effect. Effects could not easily be explained on the basis of changes in RAAS activity, natriuretic peptides or cGMP. Future studies should address to what degree ARNI affects local concentrations of natriuretic peptides, e.g. in the kidney. Here, natriuretic peptides might improve renal haemodynamics, but also exert anti-inflammatory effects.

In an earlier study in nondiabetic hypertensive rats, we observed that the dose of the NEP inhibitor given on top of an ARB is highly critical [28•]: when the NEP inhibitor was dosed too high, circulating endothelin-1 levels started to rise, and this resulted in an increase in renal sodium-hydrogen exchanger type 3 protein expression and an upregulation of constrictor vascular endothelin type B receptors. As a consequence, blood pressure rose and a beneficial effect on cardiac hypertrophy was no longer observed. Since NEP is capable of cleaving endothelin-1, these findings are not unexpected and emphasize the need to measure changes in endothelin-1 in future ARNI studies.

## Human Studies

### Effects in the General Hypertensive Population

ARNI was compared to valsartan in a proof of concept trial, to study whether neprilysin inhibition on top of ARB indeed enhances blood pressure reduction (Table 2) [25]. The study included 1328 patients with mild to moderate uncomplicated hypertension. At baseline, untreated mean sitting systolic blood pressure (SBP) averaged 155.7 mmHg and mean sitting diastolic blood pressure (DBP) averaged 99.7 mmHg. Patients with diabetes, cardiac or renal disease were excluded. Treatment with 100, 200 or 400 mg LCZ696 once daily was compared to respectively 80, 160 or 320 mg valsartan once daily (i.e. equipotent amounts of valsartan [18]), or placebo, for a treatment period of 8 weeks. Most of the treatment effect occurred in the first week after start of the study drug, and the effect was almost maximal at 4 weeks of treatment. Treatment with 200 mg LCZ696 lowered mean sitting SBP with 11.0 mmHg and mean sitting DBP with 6.1 mmHg. This is an additional reduction of –5.28 mmHg (95% CI –8.28 to –2.28) in SBP and –2.97 mmHg (95% CI –4.88 to –1.07) in DBP when compared to 160 mg valsartan. LCZ696-treated patients responded 14% more often to treatment, attested by either a blood pressure below 140/90 mmHg or more than 20 or 10 mmHg reduction in SBP or DBP, respectively. Additional ambulatory blood pressure measurements demonstrated that these reductions were maintained throughout the day, although responses were most pronounced during nighttime. These outcomes did not improve further with 400 mg LCZ696 versus 320 mg valsartan. Occurrence rate or severity of reported adverse events did not differ between LCZ696 and valsartan.

**Table 2:** Main characteristics of LCZ696-treated patients and effects of LCZ696 treatment

Study	Ruilope et al.	McMurray et al.	Solomon et al.	Ito et al.
		(PARADIGM-HF)	(PARAMOUNT)	
LCZ696-treated patients (*n*)	168	4187	149	32
Main inclusion criterium	Hypertension	HFrEF	HFpEF	CKD
Additional specifications		LVEF 29.6% (6.1)	LVEF 58% (7.3)	Stage 3 or 4
Age (years)	53 (10.2)	63.8 (11.4)	71 (9.4)	66 (9.1)
Sex (% male)	57	78	43	75
Race (% black)	8	5	NP	0
Dosage LCZ696 (mg)	200	200	200	200–400 (titration)
Dosing frequency (per day)	1	2	2	1
Duration of treatment (weeks)	8	116	36	8
Concomitant anti-hypertensive medication				
β-blockers (%)	0	93	79	0
Diuretics (%)	0	80	100	6
Mineralocorticoid receptor antagonists (%)	0	54	19	0
Calcium channel blockers (%)	0	NP	NP	38
Baseline blood pressure				
Systolic (mmHg)	156.8 (12.0)	122 (15)	136	
			(IQR 130 to 145)	151.6 (10.3)
Diastolic (mmHg)	99.9 (4.1)	NP	79	
			(IQR 74 to 85)	86.9 (10.8)
Change compared to baseline blood pressure				
Systolic (mmHg)	−11.0 (NP)**	NP	−7.5 (15.0)*	−20.5 (11.3)^$^
Diastolic (mmHg)	−6.1 (NP)*	NP	−5.1 (10.8)*	−8.3 (6.3)^$^
Main outcome	Blood pressure reduction*	CVD-mortality/HF-hospitalization reduction^#^	Left atrial volume reduction*	Blood pressure^$^ UACR reduction

Data are represented as mean (SD), unless otherwise indicated. Statistical significance was calculated using a Student’s *t* test (*P < 0.01, **P < 0.001 compared with valsartan-treated controls; ^#^ *P* < 0.001 compared with enalapril treated controls; ^$^ *P* < 0.001 compared to baseline)

*NP* not provided

### Effects in Heart Failure Populations

RAS blockade reduces cardiovascular and all-cause mortality as well as hospitalization for worsening heart failure in patients with reduced ejection fraction (HFrEF) [29]. ACEi has obtained a central position herein, since enalapril was shown to greatly improve outcome already 25 years ago [30, 31]. A recent meta-analysis of three trials that studied the combination of RAS and neprilysin inhibition in HFrEF patients demonstrated a 15% risk reduction for the composite endpoint of death or hospitalization when compared to ACEi alone [32]. Unfortunately, combination of ACEi and neprilysin inhibition (omapatrilat) is more likely to cause serious angioedema [33, 34], prompting discontinuation of drug development. Therefore, the effect of ARNI on HFrEF outcome was studied in the Prospective Comparison of ARNI With ACEi to Determine Impact on Global Mortality and Morbidity in Heart Failure (PARADIGM-HF) trial (Table 2) [35•]. This study included 8399 patients with a mean left ventricular ejection fraction (LVEF) of 29.5%. Nearly all patients continued receiving other drugs known to improve survival. Patients were randomly assigned to receive 200 mg LCZ696 twice daily or 10 mg enalapril twice daily. The trial was stopped early with a mean follow-up duration of 27 months due to a benefit in favour of LCZ696. The risk for the composite primary endpoint of cardiovascular-related death or first hospitalization for worsening heart failure was reduced by 20% in LCZ696-treated patients. In addition, all-cause mortality was reduced by 16%. Patients with lower LVEF had a 9% increased risk per five-point reduction of ejection fraction but benefitted equally from treatment when compared to patients with higher LVEF [36]. The beneficial effect on mortality is mainly due to a reduction in sudden death or death from progression of heart failure [37]. Progressive impairment of functional capacity due to nonfatal worsening of symptoms was assessed by the Kansas City Cardiomyopathy Questionnaire (KCCQ). This score improved in patients treated with LCZ696 and declined in patients treated with enalapril (mean difference between groups was 0.95 points on a scale of 1 to 100). However, patients were 34% less likely to visit the emergency room and fewer patients required intravenous drug therapy, rehospitalization or admission to an intensive care unit. Moreover, LCZ696-treated patients were 22% less likely to require surgical implantation of a left ventricular assist device (LVAD) or heart transplantation [38]. Concomitant hypertension was present in 71% of the study population. Mean SBP at baseline averaged 122 ± 15 mmHg. LCZ696 treatment lowered SBP with an additional 3.2 ± 0.4 mmHg when compared to enalapril. Consequently, LCZ696-treated patients were more likely to develop symptomatic hypotension (14 versus 9.2%). In addition, more cases of angioedema were reported (19 versus 10 patients), although this difference was not significant. Conversely, patients treated with enalapril were more likely to develop hyperkalaemia or cough. Currently, the European Society of Cardiology (ESC) recommends the use of sacubitril/valsartan instead of ACEi in ambulatory patients who remain symptomatic despite optimal therapy [39].

**Fig. 2:**
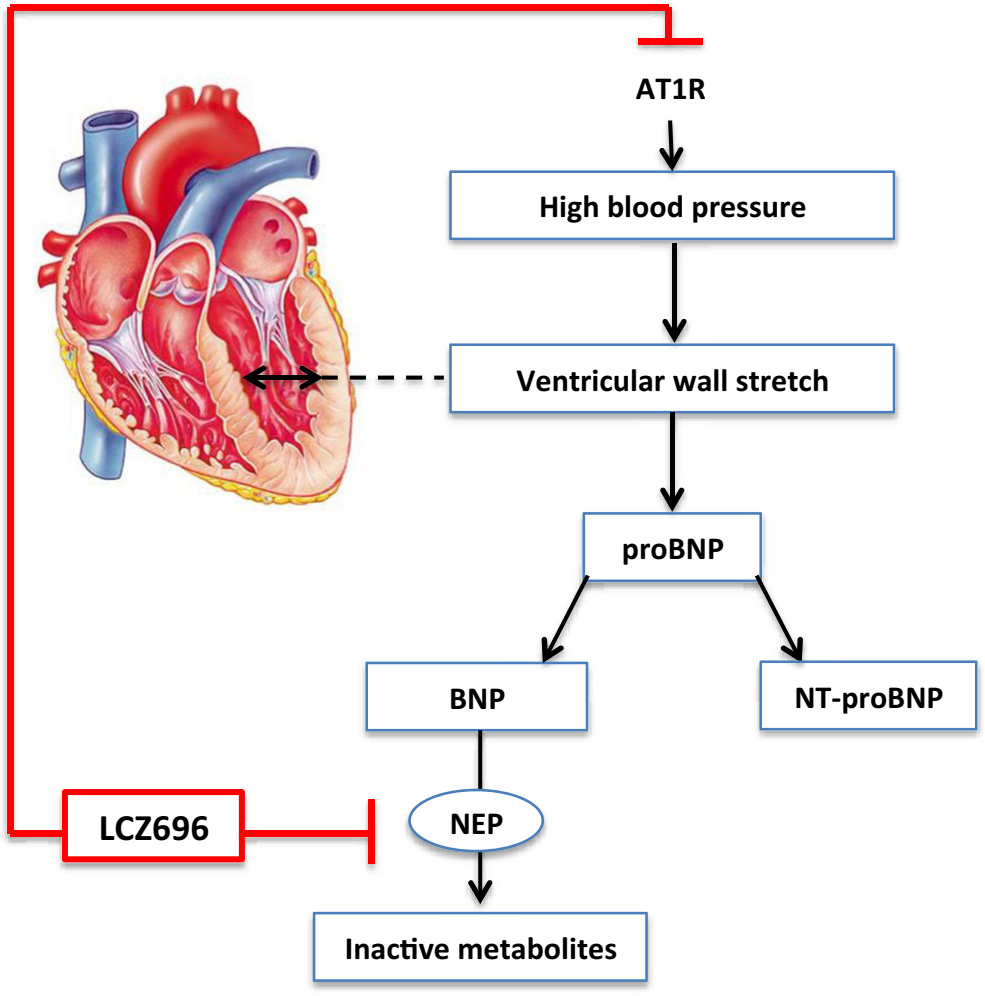
Effect of LCZ696 on BNP and NT-proBNP production. LCZ696 lowers blood pressure by simultaneous blockade of angiotensin II type 1 receptors (AT1R) and inhibition of neprilysin (NEP). Relative dominance of either effect determines BNP levels. NT-proBNP represents effects on ventricular wall stretch, as it is not degraded by neprilysin

Almost 50% of heart failure patients have a preserved ejection fraction (HFpEF). RAS inhibition is less beneficial in this group of patients. Although it reduces the risk of hospitalization due to worsening of symptoms with 12%, there are no improvements in cardiovascular or all-cause mortality [29]. Efficacy and safety of LCZ696 treatment in HFpEF patients was studied in Prospective Comparison of ARNI with ARB on Management of Heart Failure with Preserved Ejection Fraction (PARAMOUNT) (Table 2) [40]. In this study, 301 patients with a mean LVEF of 58% were included. At baseline, most patients were treated for hypertension (93.5%) and mean blood pressure averaged 136/79 mmHg. Patients were randomly assigned to 200 mg LCZ696 twice daily or 160 mg valsartan twice daily for a treatment period of 36 weeks. A significantly greater degree of blood pressure reduction was observed in LCZ696-treated patients than in valsartan-treated patients (−7.5 (±15) versus −1.5 (±16) mmHg for mean sitting SBP; −5.1 (±10.8) versus −0.34 (±11.5) mmHg for mean sitting DBP). Ventricular wall stretch induces production of pro-B-type natriuretic peptide (proBNP). N-terminal pro-B-type natriuretic peptide (NT-proBNP) is the inactive side product yielded upon cleavage of proBNP into biologically active BNP. Elevated serum NT-proBNP is a well-known marker for heart failure severity, as it is associated with increased risk of mortality and hospitalization [41]. NT-proBNP is not degraded by NEP and as such has a longer half-life than BNP. A decrease in NT-proBNP levels suggests reduced proBNP production due to reduced cardiac wall tension (Fig. 2), while an increase in BNP either suggests the opposite or might be due to NEP inhibition. In HFrEF patients, LCZ696 reduced NT-proBNP and increased BNP [38]. In HFpEF patients, LCZ696 also reduced NT-proBNP at 12 weeks of treatment [40]. At 36 weeks, this difference was no longer significant. However, at this time point, left atrial width (mean difference 0.7 mm) and volume (mean difference 4.97 mL) were reduced in favour of LCZ696. These effects were independent of blood pressure lowering [42]. Effect of ARNI treatment on mortality and hospitalization in HFpEF patients is currently being studied in Prospective Comparison of ARNI with ARB Global Outcomes in Heart Failure with Preserved Ejection Fraction (PARAGON-HF).

### Effects in Other High-Risk Populations

CKD patients are at high risk for cardiovascular morbidity and mortality, added on top of the risk of progression towards ESRD. Diabetes and hypertension are the most common causes of CKD worldwide and additionally independent risk factors for cardiovascular complications. Simultaneous presence of all three risk factors is not uncommon. However, the complex interplay between these diseases does appear to alter treatment responses. For HFrEF patients with renal function decline, regardless of cause and comorbidities, benefit of treatment with LCZ696 was equal for patients with eGFR ≥60 mL/min/1.73 m^2^ and reduced eGFR (between 30 and 60 mL/min/1.73 m^2^) [36]. However, treatment of diabetic HFrEF patients with 200 mg LCZ696 twice daily did not reduce cardiovascular and all-cause mortality when compared to valsartan, although risk for heart failure hospitalization was improved, comparable to that of nondiabetic patients [43]. Interestingly, LCZ696 reduced NT-proBNP levels particularly in HFpEF patients with diabetes, indicating a more pronounced reduction of ventricular wall tension when compared to nondiabetic patients [40]. Furthermore, LCZ696 may prevent progression of renal damage especially in patients with diabetic nephropathy. LCZ696 reduced urinary albumin-to-creatinine ratio (UACR) by 15.1% in patients with hypertension and moderate to severe renal impairment, with a greater effect in patients with macroalbuminuria than in micro- or normoalbuminuria [44•]. Conversely, a small but significant increase in UACR (2.4 to 2.9 mg/mmol) was observed in LCZ696-treated HFpEF patients [45], despite the fact that eGFR was better preserved after 36 weeks of treatment (−1.6 versus −5.2 mL/min/1.73 m^2^) [41]. Increased albuminuria in these patients might be caused by ANP-induced relaxation or anti-proliferative effects in renal mesangial cells [46, 47], in combination with impaired proximal tubular protein reabsorption [48]. No significant differences in renal outcome were found between patients with HFrEF treated with either LCZ696 or enalapril, although numerically fewer patients developed ESRD with LCZ696 (8 versus 16 patients, *P* = 0.11). These favourable outcomes might be explained by a reduction of renal tubulointerstitial fibrosis and focal segmental glomerulosclerosis, as observed in ARNI-treated diabetic, hypertensive rats when compared to ARB-treated controls [27]. Moreover, LBQ657 reduced the Ang II-stimulated increase in collagen synthesis in renal mesangial cells [49]. These effects occurred in a blood pressure-independent manner.

Despite these potential advantages in renal outcome, there may be some limitations to the use of LCZ696 in CKD patients. Sacubitrilat is primarily eliminated by the kidneys. The degree of decline in eGFR correlated with increased maximum plasma drug concentration and half-life, as the percentage of sacubitrilat excreted in urine decreased progressively. Therefore, normotensive patients with moderate to severe renal impairment more often developed orthostatic hypotension [20]. In PARADIGM-HF, blood pressure was well-controlled due to the continued use of several classes of anti-hypertensive drugs, and mean SBP at baseline averaged 122 ± 15 mmHg (Table 2) [35•]. Consequently, hypotension occurred more often in patients that were subsequently exposed to LCZ696 and was the most common reason for dose reduction, whereas a higher serum creatinine level was the main predictor of this phenomenon. This indicates that indeed normotensive patients with reduced renal function are at higher risk of developing hypotension. However, hypertension is both a cause and consequence of CKD and blood pressure is often poorly controlled in these patients [50]. Therefore, the safety and efficacy of LCZ696 was assessed in 32 Japanese patients with hypertension and CKD stage 3 (78.1%) or stage 4 (21.9%) (Table 2) [44•]. Initial treatment with 100 mg LCZ696 once daily reduced mean sitting blood pressure to 130/80 mmHg or lower in 19% of the population. The dosage in other patients was increased to either 200 mg (25%) or 400 mg (56%) LCZ696 once daily, for a total treatment duration of 8 weeks. The blood pressure-lowering effect occurred mostly in the first week of treatment and was maximal at 4 weeks. Stage 3 CKD patients had a mean blood pressure of 152.1/87.7 mmHg at baseline. With LCZ696, 60% of the patients had an adequate SBP reduction of ≥20 mmHg or values ≤130 mmHg and in 76% of the patients an adequate DBP reduction of ≥10 mmHg or values ≤80 mmHg. Mean reduction in all patients was 21.3/9.1 mmHg. LCZ696 treatment was also effective in stage 4 CKD patients. Their mean baseline blood pressure of 149.8/83.9 mmHg was reduced by 17.7/5.5 mmHg. Adequate SBP and DBP responses were obtained in 57% of the patients. No events of hypotension were reported in either CKD class, although plasma doses of LBQ657 tended to increase with decreasing renal function. The safety of LCZ696 treatment in CKD patients and effects on renal function are currently being studied in the United Kingdom Heart and Renal Protection III (HARP-III) trial [51].

### Limitations

NEP is involved in the degradation of many peptides, among which amyloid beta 1-42 (Aβ_1-42_). Intracerebral infusion of the NEP inhibitor thiorphan induces hippocampal accumulation and deposition of Aβ_1-42_ in rats, already at in vivo concentrations of 2.5 ng/mL [52]. Aggregation of Aβ_1-42_ is believed to play a critical role in the development of Alzheimer’s disease (reviewed in [53]). Blood-brain barrier penetration of LBQ657 was assessed in 17 healthy volunteers treated with 400 mg LCZ696 once daily [54]. After 2 weeks of treatment, a mean maximum drug concentration of 19.2 (±11.3) ng/mL was found in cerebrospinal fluid (CSF). There was no effect on CSF Aβ_1-42_ concentration when LCZ696-treated individuals were compared to placebo-treated controls. Differences in plasma Aβ_1-42_ concentration were not determined, although plasma Aβ_1-40_ concentration increased by 50%. These results do not dispel all concerns. CSF clearance of Aβ appears to be a minor route, as the blood-brain barrier traffics most of the brain Aβ directly out into the peripheral circulation [55]. Additionally, the measurements in placebo-treated controls demonstrate high variability of CSF Aβ_1-42_ concentration. Therefore, these results do not rule out the possibility of accumulation Aβ_1-42_ in the brain, which would go undetected by CSF measurements. Additionally, hypertension and diabetes are known to contribute to blood-brain barrier dysfunction, rendering these patients more vulnerable, as NEP inhibition might induce Aβ_1-42_ in a dose-dependent manner [56]. However, these risks might be more relevant for diseases with greater longevity than HFrEF.

## Conclusions

In summary, when compared to ARB, ARNI superiorly reduces blood pressure in uncomplicated, essential hypertension as well as in heart failure and CKD populations. When compared to ACEi, ARNI superiorly prevents death and hospitalization in HFrEF patients that already receive all other medication known to improve outcome. ARNI appears to improve renal outcome, particularly in high-risk, multi-morbid populations. Although patients with concomitant heart failure and diabetes show no reduction in mortality, they do demonstrate reverse left atrial remodeling, less heart failure hospitalization and reduced albuminuria. Preclinical data in hypertensive, diabetic rats additionally suggests blood pressure-independent improvement of renal damage, attested by decreased proteinuria and abated glomerulosclerosis. ARNI might mediate these effects by impeding activation of the proinflammatory cytokine TGF-β, which plays a central role in the fibrotic response and accumulation of collagen. This could explain the improved renal outcomes but requires further experimental study. Importantly, ARB and ARNI have not yet been compared in patients with CKD due to diabetes and hypertension, and thus the results of the HARP-III trial are anxiously awaited [51]. Until that moment, the application of ARNI as a renoprotective drug for this high-risk patient population is hampered. Moreover, sacubitrilat, the active neprilysin-inhibiting component, is eliminated by the kidney. Sacubitrilat-exposed normotensive patients with impaired renal function can therefore, due to increased exposure, develop hypotension. This is less likely to be a problem for the hypertensive population. Caution is advised regarding the risk of cerebral Aβ_1-42_ accumulation and Alzheimer’s disease in populations with a longer median survival. Finally, we need additional insight into the mechanism of action of ARNI. Although a decrease in NT-proBNP and a rise in BNP have been reported [38], this might be an assay artefact. Indeed, ARNI may promote peptide glycosylation, thereby affecting not only proBNP cleavage but also the assays of NT-proBNP and BNP: NT-proBNP would become invisible, while proBNP would additionally be detected in BNP assays [57•]. Another complicating factor is that BNP of all natriuretic peptides is the least susceptible to degradation by NEP and as such acts as an endogenous inhibitor of NEP [58]. If so, patients with elevated BNP levels already undergo NEP inhibition and might respond less or not at all to LCZ696.
